# Fast Temperature-Gradient COLD PCR for the enrichment of the paternally inherited SNPs in cell free fetal DNA; an application to non-invasive prenatal diagnosis of β-thalassaemia

**DOI:** 10.1371/journal.pone.0200348

**Published:** 2018-07-25

**Authors:** Stefania Byrou, G. Mike Makrigiorgos, Agathoklis Christofides, Ioannis Kallikas, Thessalia Papasavva, Marina Kleanthous

**Affiliations:** 1 Molecular Genetics Thalassaemia Department, The Cyprus Institute of Neurology and Genetics, Nicosia, Cyprus; 2 The Cyprus School of Molecular Medicine, Nicosia, Cyprus; 3 Department of Radiation Oncology, Division of Medical Physics & Biophysics, Dana Farber Cancer Institute, Harvard Medical School, Boston, Massachusetts, United States of America; 4 Medical Centre Fetal Medicine Department, Archbishop Makarios III Hospital, Nicosia, Cyprus; 5 Ultrasound and fetal medicine centre, Nicosia, Cyprus; Defense Threat Reduction Agency, UNITED STATES

## Abstract

**Objective:**

To develop a sensitive, specific, simple, cost-effective and reproducible platform for the non-invasive prenatal detection of paternally inherited alleles for β-thalassaemia. The development of such an assay is of major significance in order to replace currently-applied invasive methods containing inherent fetal loss risks.

**Methods:**

We present a fast Temperature-Gradient Co-amplification at Lower Denaturation Temperature Polymerase Chain Reaction (fast TG COLD PCR) methodology for the detection of the paternally-inherited fetal alleles in maternal plasma. Two single-nucleotide polymorphisms (SNPs), rs7480526 (G/T) and rs968857 (G/A) that are located on the β-globin gene cluster and exhibit a high degree of heterozygosity in the Cypriot population were selected for evaluation. Seventeen maternal plasma samples from pregnancies at risk for β-thalassemia were analysed for the selected SNPs using the novel fast *TG* COLD PCR assay.

**Results:**

Using fast TG COLD PCR, the paternally inherited allele in cell free fetal DNA was correctly determined for all the 17 maternal plasma samples tested, showing full agreement with the Chorionic Villus Sampling (CVS) analysis.

**Conclusions:**

Our findings are encouraging and demonstrate the efficiency and sensitivity of fast TG COLD PCR in detecting the minor paternally-inherited fetal alleles in maternal plasma for the development of a NIPD assay for β-thalassaemia.

## Introduction

β-hemoglobinopathies are the commonest autosomal recessive single-gene disorders worldwide[1], with β-thalassemia having a carrier frequency of about 12% in Cyprus[2]. The intronic β-globin gene mutation IVSI-110 (c.93 -21G>A) represents the 79.01% of the total β-globin gene mutation frequencies in the island[3]. At the moment, only invasive prenatal diagnosis for at risk for β-thalassemia pregnancies is performed which carries a procedure-related risk of 0.1%-0.2%[4] for induced abortion and causes thus psychological stress to the couple[5]. The discovery of fetal-origin cell-free DNA in blood circulation is of immense significance[6] for prenatal diagnostics. However, it is present in low quantities i.e. 5–20%[7,8] and in fragmented form with a prominence size of 143bp[9]. Therefore, handling such samples entails many challenges. Fetal sex determination[10,11] and fetal Rhesus status[12–14]were of the firsts Non-invasive Prenatal Diagnosis (NIPD) applications as a standard antenatal care. Due to global demand, extensive studies have been performed on Non-invasive Prenatal Testing (NIPT) for chromosomal aneuploidies that resulted in the successful translation in clinical practice using Next Generation Sequencing (NGS) technology[15–17]. NGS has enabled the introduction of the genetic analysis of recessive[18] and X-linked inherited conditions[19]. Different methods and approaches have been attempted by several groups for the NIPD of monogenic disorders[20–23]. Full Co-amplification at Lower Denaturation temperature Polymerase Chain Reaction (COLD PCR)[24–26], digital PCR[27] coupled with Relative Mutation Dosage (RMD)[28] and Relative Haplotype Dosage approach (RHDO) coupled with massively parallel sequencing[9,18,29–31]are methodologies previously used for the NIPD of β-thalassaemia. A targeted NGS approach using Single Nucleotide Polymorphisms (SNPs) has also been implemented by our group, where the paternally-inherited fetal allele was detected in maternal plasma samples and non-invasive prenatal haplotype analysis was successfully achieved in 8 families[32]. Vermeulen’s group demonstrated that NIPD for Cystic Fibrosis, Congenital Adrenal Hyperplasia and β-thalassaemia was possible using Targeted Locus Amplification based phasing with NGS [23]. Due to its high throughput and high analytical sensitivity and specificity, NGS is the method of choice in most research groups, already applied in the diagnostic setting. Nevertheless, due to its high cost and need for specialized equipment and sophisticated bioinformatics software for data analysis, its broad implementation is limited. In this study, we aimed to exploit the feasibility of the COLD PCR technology as an alternative, simple, cost-effective, time-efficient and potentially globally adopted method in enriching and detecting the paternally-inherited fetal alleles in maternal plasma. This technology will be used for the development of a reliable NIPD assay for β-hemoglobinopathies, with ultimate aim the replacement of currently-available invasive procedures.

COLD PCR preferentially amplifies minor alleles that are present in mixtures with excess major alleles[33]. This technology is based on preferential denaturation of mismatch-forming variations at critical denaturation temperature during PCR. Tc is lower than the Tm of the major alleles and is experimentally determined such that it results in differential denaturation of the minor allele population and subsequent enriched over major alleles during PCR cycling.

COLD PCR can be applied in several formats, like fast COLD PCR[33], full COLD PCR[33], Temperature-Tolerant COLD PCR[34] etc. Fast COLD PCR is suitable for enrichment of Tm-reducing variations (G: C > A: T or G: C > T: A)[35]. The melting temperature of the major (G,C containing) alleles is by default higher than the melting temperature of the minor (A,T containing) alleles, and thus when the denaturation temperature is set to an appropriate Tc, the minor alleles are being selectively denatured and amplified whereas major alleles remain substantially double-stranded and less amplified. To define the critical denaturation temperature for an efficient, repeatable and finely-tuned fast COLD PCR cycling, intensive trials and effort are required. Fast COLD PCR has been applied mostly for the enrichment of cancer mutations up until now[34,36–38].

In this study, we present a novel, modified version of fast COLD PCR applied for NIPD development for the first time; the fast Temperature-Gradient COLD PCR. This COLD-PCR approach applies a range of potential critical temperatures to enable robust and repeatable enrichment of specific minor alleles, thus bypassing the need for precise definition of a single Tc. This method can be easily applied in any laboratory since it does not require sophisticated equipment and it is highly reproducible. Its simplicity, low cost and time-efficacy distinguish it from others and render it more attainable.

Below, we present the efficiency of the methodology for the enrichment and detection of the minor paternally inherited fetal alleles in the maternal plasma of 17 pregnant β-thalassaemia carrier women at risk of having a β-thalassaemia child. This was demonstrated by using two highly heterozygous and informative SNPs located on the β-globin gene cluster[39], promoting the introduction and optimization of more SNPs than those currently applied. The analysis of multiple SNPs in combination with haplotype analysis and allelic phasing has as an ultimate scope the determination of the fetal genotype using maternal plasma. SNPs for which the parents are both homozygous but for different alleles can be used to confirm the presence of fetal DNA within maternal plasma. In this approach, the informative SNPs are those for which the mother is homozygous A/A and the father is heterozygous A/B, thus enabling the determination of the phase of the paternally-inherited fetal allele. In that way, 50% of the cases can benefit, i.e. when the mutation free paternal allele is inherited the fetus is either mutation free or carrier of the mutation. Therefore the fetus is unaffected and as a result invasive procedures are avoided. In cases where the fetus inherits the affected paternal allele, it is either carrier or affected and hence invasive procedures are required.

Here, we present the fast TG COLD PCR approach as being an easy, rapid, cost-effective and easily-adopted methodology for the detection of the paternally inherited allele in cell-free fetal DNA from maternal plasma as an application for the NIPD of β-thalassaemia as well as for other monogenic diseases.

## Materials and methods

### Ethical approval

The study was approved by the Cyprus National Bioethics Committee (EEBK/EΠ/2017/06) and the Ethics Committee of the Cyprus Institute of Neurology and Genetics. All participants were β-thalassaemia carrier couples being at a risk for having a child with β-globin gene disorder, who were referred to our laboratory for routine prenatal diagnosis. The couples were counseled on the 11^th^ week of pregnancy about the goals and benefits of this study and informed about the sampling procedure in case they agree in participating. A written consent form describing the aim of the study, the participants’ rights and the confidentiality of the principal investigator was given to the couples to be read carefully and signed only after their consent. Hence, all participants gave informed written consent (EEBK003/EΠ/2017/06). The research has been complied with all the relevant national regulations and in accordance the tenets of the Helsinki Declaration.

### Sample collection

Approximately 9 ml of peripheral blood samples were collected into EDTA-containing tubes from seventeen pregnant women between the 10^th^ and 11^th^ week of gestation at risk for β-thalassemia in their newborn. Only singleton pregnancies were analyzed. Peripheral Blood was collected from the partner and their parents.

### DNA extraction

The isolation of maternal plasma from peripheral blood was performed as previously described[32]. Cell-free DNA was extracted from 1ml of maternal plasma using QIAamp Circulating Nucleic Acid Kit according to the manufacturer’s instructions (Qiagen GmbH, Hilden, Germany). Genomic DNA was extracted using the Puregene Blood Core Kit C according to the manufacturer’s instructions (Qiagen Sciences, Germantown, MD, USA).

### SNP selection and genotyping

A panel of 49 SNPs located on the β-globin cluster on chromosome 11 and exhibiting high degree of heterozygosity (>6%) in the Cypriot population was determined through a study performed by Papasavva et al[39]. Two informative SNPs that follow the requirement of fast TG COLD PCR for allelic Tm differences, i.e. rs7480526 (G/T) and rs968857 (G/A) were selected from the predetermined SNP panel for analysis.

### Primer design

All primer sequences (Table 1) were designed using the reference sequence of hemoglobin gene locus based on the human genome assembly GRCh37 with accession number NG_000007 from the NCBI database via the Vector NTI software. Primer sequences were assessed to avoid homology to other loci in the genome by using the Basic Local Alignment Search Tool (BLAST) (https://blast.ncbi.nlm.nih.gov/Blast.cgi).

**Table 1:** Primer sequences and fragment sizes for pre-amplification and fast Temperature-Gradient COLD PCR.

SNP ID number	rs7480526 (g/t)	rs968857 (g/a)
**Fragment size (bp)**	102	153
**PCR primer (forward, 5’-3’)**	GTGAGTCTATGGGACGCTTG	GTGATATCTTAGTATTTATAGGTCATGAG
**PCR primer (reverse, 5’-3’)**	TCCCATTCTAAACTGTACCC	CTGATATAACTAATAAACTGACTTCTGA
**Sequencing primer (5’-3’)**	CTTTCCCCTTCTTTTCTATG	TAGGTCATGAGGTTCCTTCC

### Determination of the critical temperature, Tc

For efficient enrichment via fast COLD PCR, the critical temperature Tc has to be defined with a precision of ±0.3°C[40]. To determine precisely the Tc for each amplicon, we first performed High Resolution Melting (HRM) curve analysis on the Rotor Gene Q (Qiagen, Hilden, Germany) to define the Tm of each amplicon using genomic DNA samples. Artificial mixtures of genomic DNA that mimic the fetal-maternal relationship were then created and consisted of 95% homozygous major allele and 5% homozygous minor allele. Gradient fast COLD PCR with a series of temperatures lower than the Tm of the major allele was then carried out using these samples, until the temperature was low enough that no PCR product was produced. Then, the lowest temperature that yielded a PCR product was set as the Tc, as also reported by Galbiati et al[26]. Using this initial approach we observed suboptimal reproducibility, despite accurate determination of the Tc. Accordingly, to obtain a reproducible enrichment of the minor allele for each SNP, after an initial amplification with conventional PCR, we developed the fast TG COLD PCR that uses a gradual increase of the denaturation temperature during thermo-cycling. The denaturation temperature is incrementally increased in three or four temperature steps accordingly, with a window of 0.5 °C for SNP rs968857 and 0.6 °C for SNP rs7480526. In this way, minor temperature differences in thermocycler cells are overcome, since the temperature spans a substantial range and encompasses the appropriate Tc value, while at temperatures lower than Tc there is no PCR product generated during cycling.

### Fast Temperature-Gradient COLD PCR

Before applying fast TG COLD PCR, we perform five rounds of conventional PCR to generate the appropriate template on which fast TG COLD-PCR will be applied. For the conventional PCR, we used 25ng of genomic DNA or 10μl of cell-free DNA from maternal plasma as a template in a reaction volume of 25μl that contained 1 x PCR buffer, 1.25mM dNTPs, 10μM set of primers and one unit of AmpliTaq Gold DNA polymerase (Applied Biosystems by Roche Molecule Systems Inc., Branchburg, NJ, USA).

The cycling conditions for conventional PCR were as follows: 95 °C for ten min; 35 cycles for genomic DNA or 40 cycles for cell-free DNA at 95 °C for 30 sec, 56 °C for 30 sec, 72 °C for 30 sec; and 72 °C for seven min.

The PCR products were then diluted 1000-fold and 2μl were used as template for the fast TG COLD PCR, as described above. All reactions were performed using the Applied Biosystems Veriti 96 Fast Thermal Cycler (Applied Biosystems, Foster City, USA).

**Fig 1:**
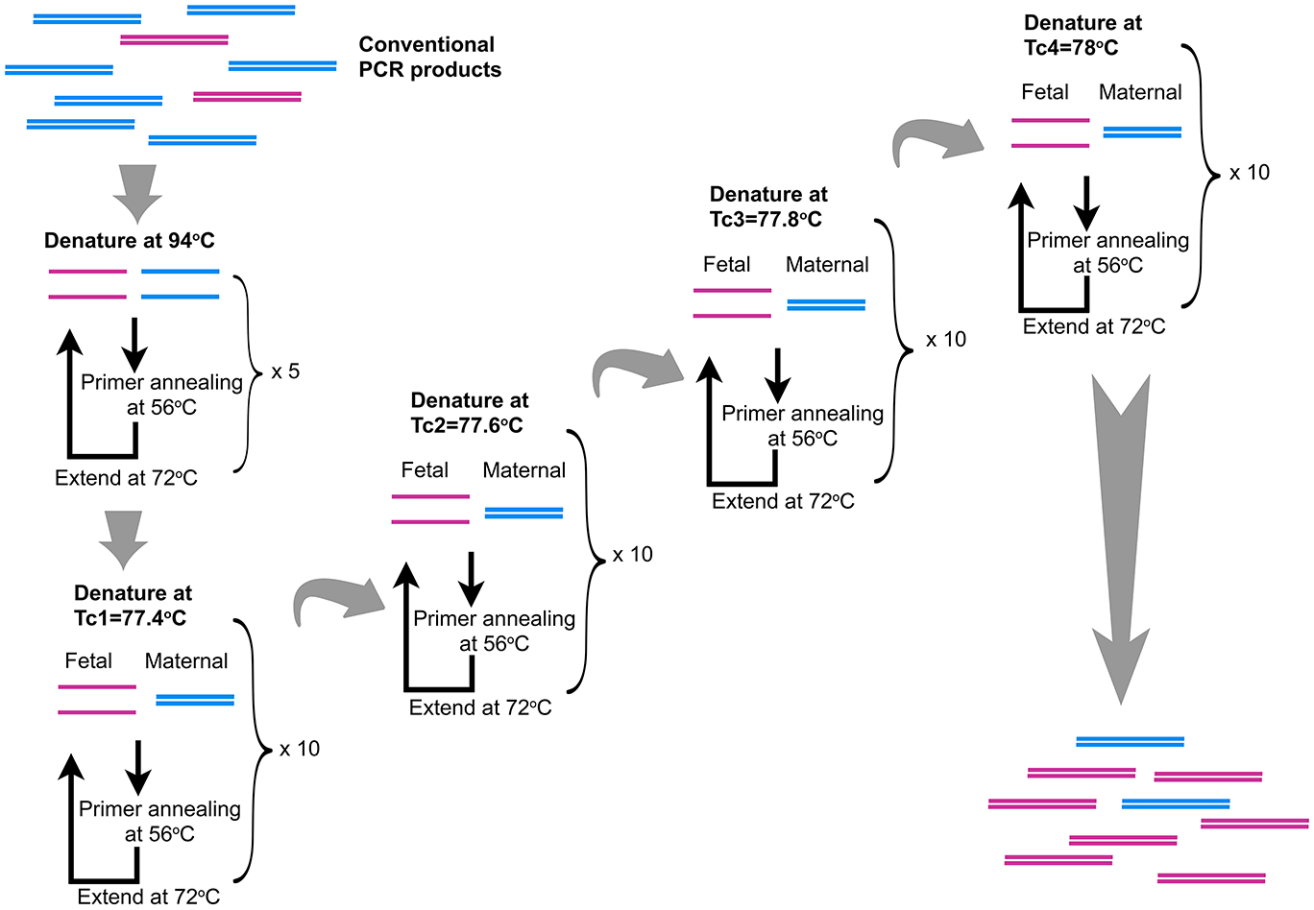
Fast Temperature-Gradient COLD PCR for rs7480526. The input conventional PCR products resulted from spiked genomic DNA amplification consisting of 95% maternal alleles (blue) and 5% fetal alleles (red). First, the PCR products are subjected to a number of cycles of regular PCR to produce an initial pool of target amplicons. Next, the denaturation temperature is set to the first Tc, which is lower than the Tm of the maternal allele. Since the melting temperature of the fetal alleles is lower than that of the maternal alleles, fetal alleles get substantially denatured whereas the maternal alleles remain substantially double-stranded. Then we decrease the temperature for primer annealing and extension. Next, we move on to the second critical temperature (Tc2) and repeat the same procedure for ten cycles. In that way we span a range of three (SNP rs968857) or four (SNP rs7480526) critical temperatures and so we assure the preferential enrichment of the minor fetal allele and increase its abundance within the final product.

The optimized fast TG COLD PCR for rs7480526 consisted of an initial step of denaturation at 94 °C for 10s, followed by five cycles of denaturation at 94 °C for 30 sec, annealing at 56 °C for 30 sec and extension at 72 °C for 30 sec. Next, ten cycles of denaturation at Tc1 = 77.4 °C for 30 sec, annealing at 56 °C for 30 sec and extension at 72 °C for 30 sec were performed. Another ten cycles of denaturation at a successive Tc (Tc2) that was 0.2°C higher than Tc1 followed by annealing and extension at the same conditions; this was followed by Tc3 until reaching the final Tc4 (Fig 1). In the end, final extension step at 72 °C for seven minutes is carried out. The Tcs for SNP rs7480526 and SNP rs968857 are presented in Table 2.

**Table 2:** Critical temperatures of SNP rs7480526 and SNP rs968857.

rs7480526 (g/t)	rs968857 (g/a)
^†^Tc1 = 77.4 °C	Tc1 = 77.3 °C
Tc2 = 77.6 °C	Tc2 = 77.5 °C
Tc3 = 77.8 °C	Tc3 = 77.8 °C
Tc4 = 78.0 °C	

^†^Critical temperature

### Sanger sequencing

The fast TG COLD PCR products were purified by EXO-SAP (New England Biolabs, Massachusetts, USA) digestion and cycle sequenced using BigDye Terminator v1.1, cycle sequencing kit (Applied Biosystems, Foster City, USA). The sequencing reactions, after dye removal were analyzed on an automated Genetic Analyzer (Applied Biosystems 3130Xl, Foster City, USA).

### Statistical analysis

Statistical analysis was performed using the R programming language (version 3.4.2), particularly for the calculation of sensitivity, specificity and confidence intervals. Specifically, the exact binomial test was utilised for the calculation of 95% confidence intervals (95% CI), using the *binom*.*test* function in R.

## Results

### Fast COLD PCR

**Fig 2:**
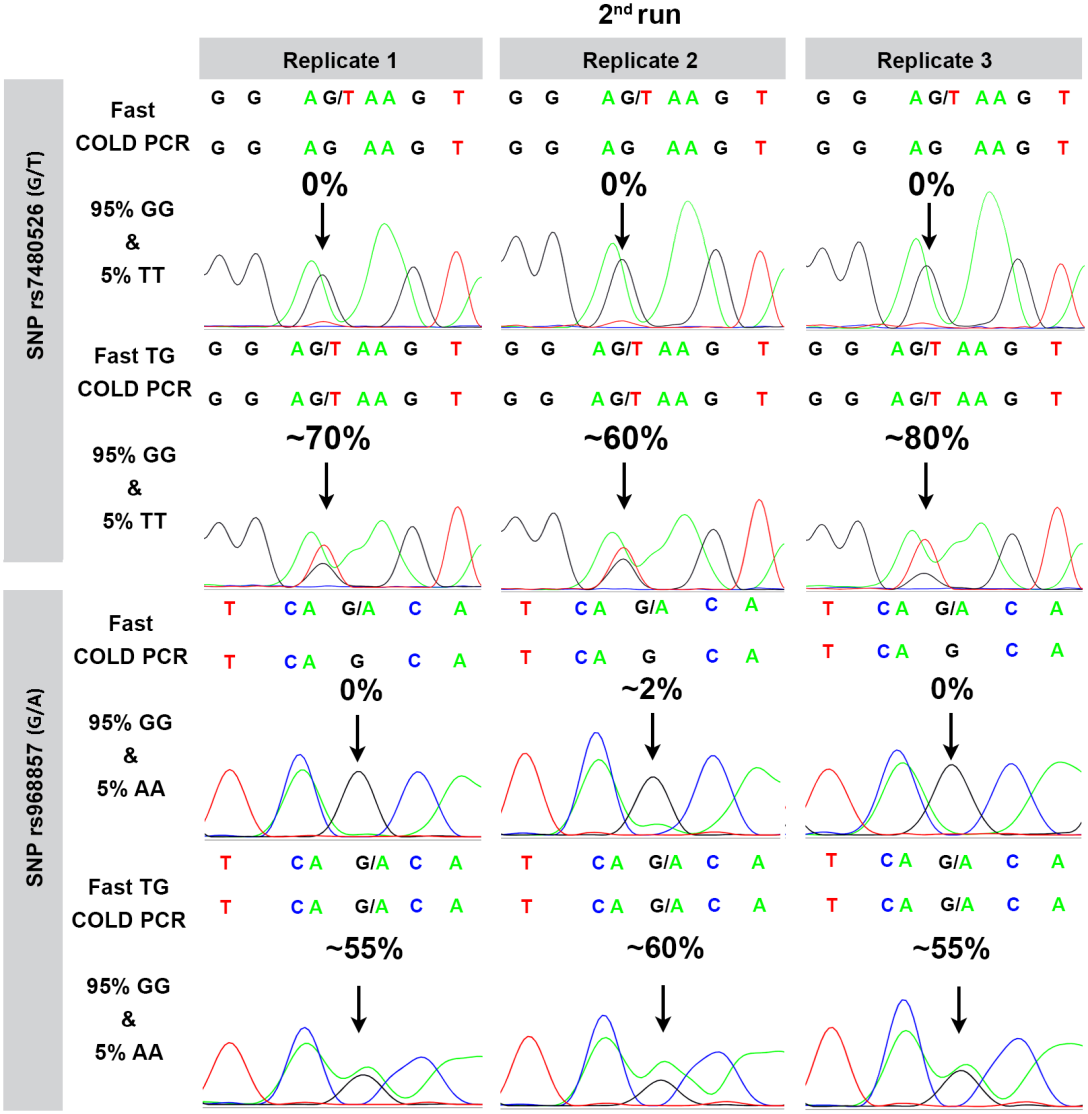
Electropherograms of spiked genomic DNA for SNPs rs7480526 and rs968857 using fast-COLD-PCR and fast-TG-COLD-PCR. The top sequence above each electropherogram presents the reference sequence based on the GRCh37 genome assembly. Enrichment of the minor allele is observed using the fast TG COLD PCR whereas non enrichment is observed with fast COLD PCR for the same samples.

The initial attempts on fast COLD PCR using pre-amplified genomic DNA spikes demonstrated promising enrichment efficiency; however, the results were not satisfactory in terms of reproducibility (Fig 2). This outcome led us to pursuit alternative solutions and resulted in the optimization of a novel COLD PCR application; the fast TG COLD PCR.

### Determination of fast Temperature-Gradient COLD PCR specificity and reproducibility

In order to evaluate the reproducibility and specificity of our fast TG COLD PCR protocols in the prenatal diagnostics setting, we used spiked genomic DNA samples that mimic the fetal-maternal relationship. Since our experiments were performed using the fast TG COLD PCR protocol which requires Tm-reducing variations, spikes were generated to fulfil this criterion. Hence, the major allele was G for both SNPs and the minor allele was T for SNP rs7480526 and A for SNP rs968857. All genomic DNA spikes along with genomic DNA homozygous controls were analyzed in triplicate, three consecutive times for SNP rs7480526 (G/T) and SNP rs968857 (G/A).

Enrichment of the minor allele T from an original 5% to about 60–80% has been accomplished in all replicates of spiked samples during genomic DNA analysis for SNP rs7480526. The three replicates of the second run are demonstrated in Fig 2. No false positive enrichment was obtained in control reactions of homozygous G and homozygous T genomic DNA samples, thus indicating the specificity of the method (data not shown). As for SNP rs7480526, enrichment of the minor allele A from 5% to nearly 60% has been also observed in all replicate genomic DNA spiked samples analyzed for SNP rs968857 and the three replicates of the second run are also shown in Fig 2.

### Fast Temperature-Gradient COLD PCR on maternal plasma

The optimized assay was then assessed on maternal plasma samples. In total, seventeen maternal plasma samples from pregnancies at risk for β-thalassemia were analyzed; eight for SNP rs7480526 (G/T) and nine for SNP rs968857(G/A). For the assessment of specificity, reliability and reproducibility of the assay, reactions were carried out again in triplicate and repeated independently 3 times. A retrospective analysis was performed on the corresponding Chorionic Villi Sample (CVS) to determine the expected genotype of the fetus.

**Fig 3:**
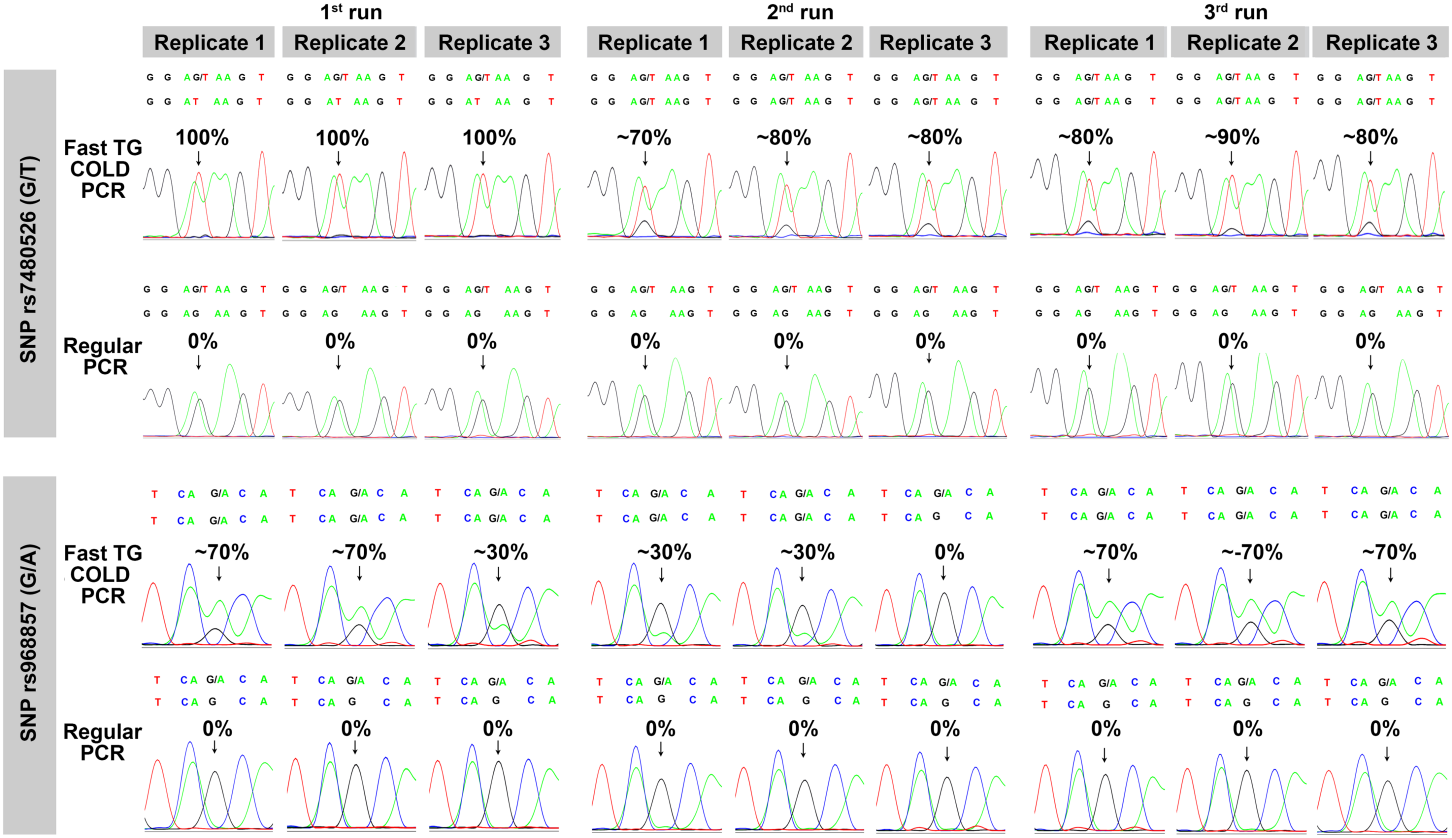
Electropherograms of maternal plasma of families 182, 197 for rs7480526, rs968857 respectively, after fast-TG-COLD-PCR/conventional-PCR. Three runs are indicated, 3 replicates per run, 9 reactions total. The top sequence above each electropherogram presents the reference sequence based on the GRCh37 genome assembly. Enrichment of the paternally inherited minor allele is observed in all replicates for SNP rs7480526 and in 8 out of 9 reactions for SNP rs968857 with fast TG COLD PCR as opposed to no enrichment with conventional PCR.

Regarding SNP rs7480526, enrichment of the minor allele T at approximately 30–100% was achieved after fast TG COLD PCR, while after conventional PCR the minor allele was undetectable. For family 179, no minor allele is present in the maternal plasma since the fetus has the same genotype with the mother. In this case, no false positive enrichment has been observed which confirms the specificity of our assay (data not shown). Significant enrichment of the minor allele A at nearly 30–80% was observed for SNP rs968857, whereas allele A was completely masked during conventional PCR. Furthermore, no other allele was detected in the maternal plasma samples for families 173, 177 and 185. This result is concordant with the CVS analysis since the fetuses of those families have the same genotype with the mother, indicating that the fetus inherited the G allele from the father (data not shown). Fig 3 demonstrates all the reactions performed with fast TG COLD PCR and regular PCR for the analysis of family 182 for SNP rs7480526 and family 197 for SNP rs968857.

### Determination of the paternally inherited allele in the cell free fetal DNA sample

The determination of the paternally inherited allele in the fetus based on cell-free fetal DNA isolated from the maternal circulation was performed for seventeen maternal plasma samples using two SNPs. Nine reactions were performed for each maternal plasma sample, in 3 separate runs, three replicates in each run; with exception for the ones with limited quantity. To determine the paternally inherited allele unambiguously, we set a threshold of 7/9 or 5/6 replicates being in agreement. The above threshold was set based on the guidelines of the accredited NIPD tests for fetal Rhesus and fetal sex determination that are implemented in our routine clinical practice since 2009[12].

**Fig 4:**
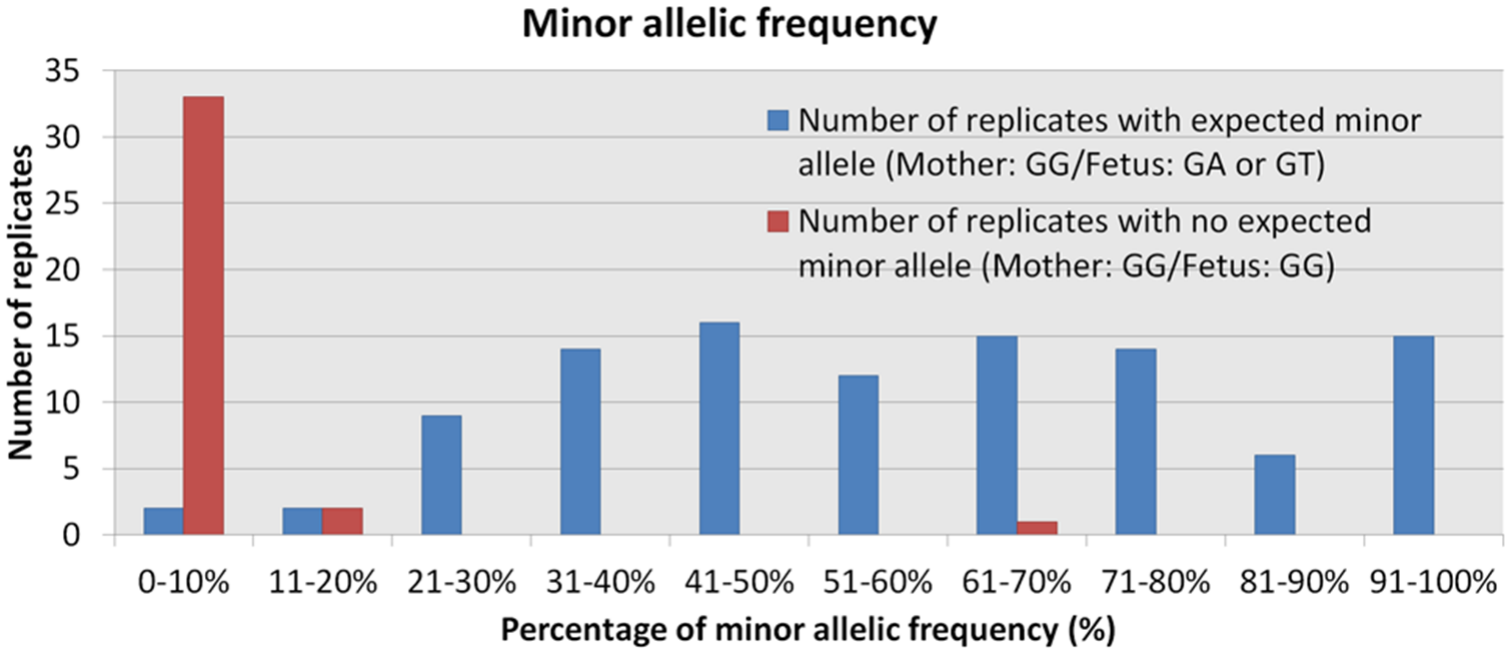
Minor allelic frequency of the 17 maternal plasma samples analyzed for all the 141 replicates. Blue columns: replicate samples with expected minor allele (Mother: GG/ Fetus: GA or GT). A minor allelic frequency distribution between 21–100% was observed in 101 out of the 105 replicates. A minor allelic frequency between 0–10% was observed in 2 out of 105 replicates and 11–20% in 2 out of 105 replicates. Red columns: replicate samples with no expected minor allele (Mother: GG/ Fetus: GG). A minor allelic frequency below 10% was observed in 33 out of the 36 replicates. A minor allelic frequency between 11–20% was observed in 2 out of 36 replicates and 61–70% minor allelic frequency in 1 out of 36 replicates.

In this study, a total of 141 replicate plasma samples have been analyzed; 105 replicates with expected minor allele (Mother: GG/ Fetus: GA or GT) and 36 replicates with no expected minor allele (Mother: GG/ Fetus: GG) (Fig 4). A minor allelic frequency distribution of 21–100% was observed in 101 out of the 105 replicates (96.2%) with expected minor allele. A minor allelic frequency of 0–10% was observed in two out of 105 replicates (1.9%) whilst in two other replicates out of the 105 (1.9%), a minor allelic frequency of 11–20% was observed. A minor allelic frequency below 10% was observed in 33 out of the 36 replicates (91,7%) of no expected minor allele. A minor allelic frequency of 11–20% was observed in two out of 36 replicates (5.5%) and 61–70% minor allelic frequency was observed in one out of 36 replicates (2.8%). Based on these results, the enrichment of the minor allele at 21% and more was set as the threshold which the results were considered as true positives. When an enrichment of 0–10% was observed, the result was considered as negative, while from 11–20% enrichment, results were classified as non-determined (ND). Overall, out of 141 replicates, 101 true positives and 33 true negatives were observed. Four non-determined results have been seen while 2 false negatives and one false positive have been observed. Nevertheless, the analysis of more samples using the fast TG COLD PCR will specify the exact level of enrichment and the number of replicates as well as the percentage of those needed for being in agreement to reliably deduce the paternal inheritance.

For SNP rs7480526, we analyzed eight families and detected successfully the paternally-inherited fetal allele in all of them. However, a false negative result was observed only in maternal plasma sample 205 with no minor allele enrichment (Table 3). Ambiguous results were observed sporadically, where the paternally-inherited fetal allele was not clearly detected after Sanger sequencing, having an enrichment of approximately 15%; thus characterized as non-determined results. This effect was noticed only in four instances, i.e. in two replicates of maternal plasma sample 179 and in one replicate of maternal plasma samples 186 and 205 (Table 3).

**Table 3:** Maternal plasma analysis results of SNP rs7480526 and SNP rs968857. Three runs and three replicate reactions per run for eight and nine maternal plasma samples respectively.

			1^st^ run			2^nd^ run			3^rd^ run			
	Family number	CVS^†^ analysis	Rep. 1	Rep. 2	Rep. 3	Rep.1	Rep.2	Rep.3	Rep.1	Rep.2	Rep.3	MP^‡^ analysis
**SNP rs7480526 (g/t)**	**Fam. 111** M^¥^:gg F^¤^:tt	gt	gt	gt	gt	gt	gt	gt	gt	gt	gt	gt
	**Fam. 167** M:gg F:gt	gt	gt	gt	gt	gt	gt	gt	Run out of sample			gt
	**Fam. 179** M:gg F:gt	gg	gg	gg	gg	gg	gg	gg	gg	ND^§^	ND	gg
	**Fam. 182** M:gg F:tt	gt	gt	gt	gt	gt	gt	gt	gt	gt	gt	gt
	**Fam. 186** M:gg F:tt	gt	gt	gt	gt	gt	gt	gt	gt	ND	gt	gt
	**Fam. 194** M:gg F:gt	gt	gt	gt	gt	gt	gt	gt	gt	gt	gt	gt
	**Fam. 199** M:gg F:gt	gt	gt	gt	gt	gt	gt	gt	gt	gt	gt	gt
	**Fam. 205** M:gg F:gt	gt	gt	gt	gt	gt	gt	gt	ND	gt	gg	gt
**SNP rs968857 (g/a)**	**Fam. 162** M:gg F:ga	ga	ga	ga	ga	ga	ga	ga	Run out of sample			ga
	**Fam. 165** M:gg F:ga	ga	ga	ga	ga	ga	ga	ga	Run out of sample			ga
	**Fam. 173** M:gg F:ga	gg	gg	gg	gg	gg	gg	ga	gg	gg	gg	gg
	**Fam. 177** M:gg F:ga	gg	gg	gg	gg	gg	gg	gg	gg	gg	gg	gg
	**Fam. 185** M:gg F:ga	gg	gg	gg	gg	gg	gg	gg	gg	gg	gg	gg
	**Fam. 190** M:gg F:ga	ga	ga	ga	ga	ga	ga	ga	ga	ga	ga	ga
	**Fam. 197** M:gg F:ga	ga	ga	ga	ga	ga	ga	gg	ga	ga	ga	ga
	**Fam. 199** M:gg F:ga	ga	ga	ga	ga	ga	ga	ga	Run out of sample			ga
	**Fam. 203** M:gg F:ga	ga	ga	ga	ga	ga	ga	ga	ga	ga	ga	ga

^**†**^Chorionic Villi Sample,

^**‡**^Maternal Plasma,

^§^Not determined,

^¥^Mother,

^¤^Father

For SNP rs968857, nine maternal plasma samples were examined. Out of nine replications in three independent experiments, one false positive result was detected in maternal plasma sample 173 with a ~70% minor allele enrichment and one false negative result in plasma sample 197 with undetectable levels of the minor allele (Table 3). Following the determination approach described above we conclude that the paternal allele was detected and differentiated in 9/9 samples.

The accuracy, the sensitivity and the specificity of our novel methodology were calculated based on the total number of replicates performed in this study as well as based on the final diagnostic result. More specifically, we obtained an accuracy of 97.8% (95% CI: 92.7, 99.2), with a sensitivity of 98.1% (95% CI: 93.2, 99.8) and a specificity of 97.1% (95% CI: 84.7, 99.9) for all replicates and an accuracy of 100% (95% CI: 80.5, 100), with a sensitivity of 100% (95% CI: 75.3, 100) and a specificity of 100% (95% CI: 39.7, 100) for final diagnostic interpretation.

Overall, we have successfully detected the paternally-inherited fetal allele in all 17 maternal plasma samples analyzed. Our results were in complete concordance with the CVS analysis performed previously in our laboratory for diagnostic purposes (Table 3).

## Discussion

In this work we have demonstrated the efficiency of a modified, novel version of fast COLD PCR; the fast TG COLD PCR. This technology has been optimized and applied by our group for the enrichment of the paternally-inherited fetal alleles in the maternal plasma samples of pregnancies at risk for β-thalassaemia, using two high-heterozygosity and informative SNPs located on the β-globin cluster. As implementation of a high number of SNPs is recommended for a reliable and accurate result, this study provides support for the introduction of more SNPs in the panel using this methodology. By using more SNPs, non-invasive haplotyping is encouraged and should be feasible. Fast TG COLD PCR has several advantages in that it is sensitive, rapid and inexpensive, without requiring sophisticated and specialized laboratory equipment or complicated bioinformatics analysis like NGS and Droplet Digital PCR. Variations in equipment and reagents between different laboratories that adopt fast COLD PCR result in enrichment differences and reproducibility difficulties. This obstacle can be moderated by implementing fast TG COLD PCR which overcomes the requirement for strict temperature control and creates a critical denaturation temperature window for robust minor allele enrichment.

Our attempts on using COLD PCR started with implementing fast COLD PCR. The preliminary results were satisfactory and with excellent enrichment efficiency but with suboptimal reproducibility. We assume that this problem is attributed to the fact that we were operating fast COLD PCR at the limit between amplification and non-amplification, and due to well-to-well thermocycler variations. Hence, we started a systematic series of trials to determine the optimal critical denaturation temperature for each SNP and obtain repeatable results, but with limited success. In 2011, Galbiati et al., developed a full COLD PCR assay for the NIPD of β-thalassemia by enriching two paternally-inherited mutations[26]. To ensure a successful enrichment of the mutant allele at least once, they used eight replicates, each with a different and minimally decreased Tc, starting from an upper Tc limit[26]. In order to obtain increased reproducibility for the enrichment of cancer mutations, others have applied two rounds of fast COLD PCR[41].

Our own efforts resulted to the modification of the fast COLD PCR technology and the development of the fast TG COLD PCR protocol. In that way, we achieved remarkably high minor allelic frequencies with reproducible enrichment, as opposed to the results obtained from fast COLD PCR trials. However, among 141 total reactions we observed one false positive and two false negatives as well as four reactions with ambiguous outcome. False positives can appear because of contamination or PCR errors at early PCR cycles, which are then enriched during COLD-PCR. On the other hand, false negatives and ambiguous results could be attributed to inefficiency of cffDNA amplification. Due to the lower mean length of cffDNA longer amplicons are less adequate for the amplification of cffDNA. When this is also coupled with low fetal fraction, it could lead to complete lack of amplification.

This pinpoints the importance of having a high number of replicates in each PCR session when handling sensitive samples such as cell-free fetal DNA in order to deduce a reliable result.

One limitation of fast TG COLD PCR is the restriction to enriching only Tm-reducing variations. This can be potentially overcome if one adopts the recently published approach “Nuclease-Assisted Minor–Allele enrichment with Probe-overlap (NaME-PrO)”[42]. This method is based on the utilization of a double-strand-DNA-specific nuclease, which requires fully matched template. For each DNA target, a pair of overlapping probes is designed, complementary to the wild type DNA. After denaturation, probe binding and upon addition of the nuclease, preferential digestion of wild-type DNA is enabled, leaving the mismatched strands undigested. Hence, subsequent amplification favors and enriches mutated alleles.

Furthermore, the proposed method will provide a diagnosis in 50% of cases since it determines the paternally-inherited fetal allele which differs from the maternal genome. In case the fetus has inherited the mutation free paternal allele, is hence unaffected but of unknown carrier status. In the event that the fetus has inherited the mutant paternal allele, is therefore of high risk and requires further testing to determine if it is a carrier or affected. This could be implemented either invasively or via more sophisticated NIPD technologies that can also determine the maternally-inherited fetal allele, like NGS[23] or droplet digital PCR[27]. Still, fast TG COLD PCR remains an easy, cost-effective, non-invasive and reliable method that allows avoidance of invasive testing in 50% of the cases where the mutation free paternal allele is inherited, classifying and directing the rest of the cases to NGS or digital PCR for the maternally inherited allele determination.

Moreover, the fact that our approach provides the base for haplotyping, indicates the requirement of genotyping parents and conducting allelic phasing, which is not always possible to do. This can be overcome by chromosomal phasing using digital PCR; a method that bypasses the need for other family members’ genetic material and performs genotyping and allelic phasing on single DNA molecules [43].

Besides autosomal-recessive disorders, fast TG COLD PCR could also have applicability in testing other single gene disorders, autosomal-dominant and *de novo* conditions based on paternal mutation exclusion. In that case, it would provide a more cost-effective solution for NIPD than the currently-used ddPCR and NGS-based assays, making prenatal diagnosis more accessible to families of low economic status.

In summary, this study demonstrated that the detection of paternally inherited fetal alleles in the maternal plasma is feasible using this simple and easily adapted methodology. The method can be coupled with other methods such as NGS or digital PCR for the complete non-invasive prenatal diagnosis for β-thalassaemia.
